# Assessment of Wind and Solar Hybrid Energy for Agricultural Applications in Sudan

**DOI:** 10.3390/en15010005

**Published:** 2021-12-21

**Authors:** Zafar A. Khan, Muhammad Imran, Abdullah Altamimi, Ogheneruona E. Diemuodeke, Amged Osman Abdelatif

**Affiliations:** 1 Department of Electrical Engineering, Mirpur University of Science and Technology, Mirpur A.K. 10250, Pakistan; 2 College of Engineering and Physical Sciences, Mechanical, Biomedical and Design Engineering, Aston University, Birmingham B4 7ET, UK; m.imran12@aston.ac.uk; 3 Department of Electrical Engineering, College of Engineering, Majmaah University, Al-Majmaah 11952, Saudi Arabia; a.altmimi@mu.edu.sa; 4 Energy and Thermofluid Research Group, Department of Mechanical Engineering, Faculty of Engineering, University of Port Harcourt, Port Harcourt 500102, Nigeria; ogheneruona.diemuodeke@uniport.edu.ng; 5 Department of Civil Engineering, Faculty of Engineering, University of Khartoum, P.O. Box 321, Khartoum 51111, Sudan; Amged.Abdelatif@uofk.edu

**Keywords:** hybrid renewable energy, techno-economic optimization, net present cost, HOMER Pro^®^

## Abstract

In addition to zero-carbon generation, the plummeting cost of renewable energy sources (RES) is enabling the increased use of distributed-generation sources. Although the RES appear to be a cheaper source of energy, without the appropriate design of the RES with a true understanding of the nature of the load, they can be an unreliable and expensive source of energy. Limited research has been aimed at designing small-scale hybrid energy systems for irrigation pumping systems, and these studies did not quantify the water requirement, or in turn the energy required to supply the irrigation water. This paper provides a comprehensive feasibility analysis of an off-grid hybrid renewable energy system for the design of a water-pumping system for irrigation applications in Sudan. A systematic and holistic framework combined with a techno-economic optimization analysis for the planning and design of hybrid renewable energy systems for small-scale irrigation water-pumping systems is presented. Different hybridization cases of solar photovoltaic, wind turbine and battery storage at 12 different sites in Sudan are simulated, evaluated, and compared, considering the crop water requirement for different crops, the borehole depth, and the stochasticity of renewable energy resources. Soil, weather, and climatic data from 12 different sites in Sudan were used for the case studies, with the key aim to find the most robust and reliable solution with the lowest system cost. The results of the case studies suggest that the selection of the system is highly dependent on the cost, the volatility of the wind speed, solar radiation, and the size of the system; at present, hybridization is not the primary option at most of sites, with the exception of two. However, with the reduction in price of wind technology, the possibility of hybrid generation will rise.

## 1. Introduction

With the technological developments, the drive for renewable energy has intensified the use of renewable energy sources (RES), particularly wind and solar photovoltaic (PV) generation. The use of renewable energy and hybridization is taking new shapes, but PV and wind energy tend to be the center point of renewable energy hybridization [1,2]. This is mainly due to the aim of the reduction of greenhouse gas emissions, but the increased penetration of RES is also due to the dropping prices of the renewable energy sources. The technological advancements in the PV [3] and wind generation systems have been formidable. The global cumulative installed capacity of all solar PV (utility scale and rooftop) increased from 42 GW in 2010 to 714 GW in 2020 [4]. Similarly, the on-shore wind cumulative installed capacity grew from 178 GW to 699 GW between 2010 and 2020 [4]. The increased generation capacity of wind and PV generation is a result of the decline in prices from USD 4731/kW to USD 883/kW for PV, and from USD 1971/kW to USD 1355/kW between 2010 and 2020 [4].

Although there has been a precipitous decline in the cost of RES, many countries are struggling to adopt these technologies due to a number of reasons, ranging from the financial resources to the technical capability for design, installation, and maintenance. This is particularly common in developing countries, and Sudan is one such country. The bulk of Sudan’s energy needs are met by using biofuels as key sources of energy, which is supported by oil and hydroelectric generation. The bulk of electricity in Sudan is generated by hydroelectric generation, and this is mainly due to the hydroelectric project completed after 2009, where hydroelectric power generation overtook oil-based generation [5]. Despite having abundant sunlight and high winds, almost 46% of the population do not have access to electricity [6]. The international community have provided support to Sudan during the years 2010 to 2012 to promote renewable energy, but the support could not be sustained for long. In year 2020, 90% of renewable electricity was produced from hydro-electric generation, 9% from biofuels, and only 1% from solar PV [7].

**Figure 1:**
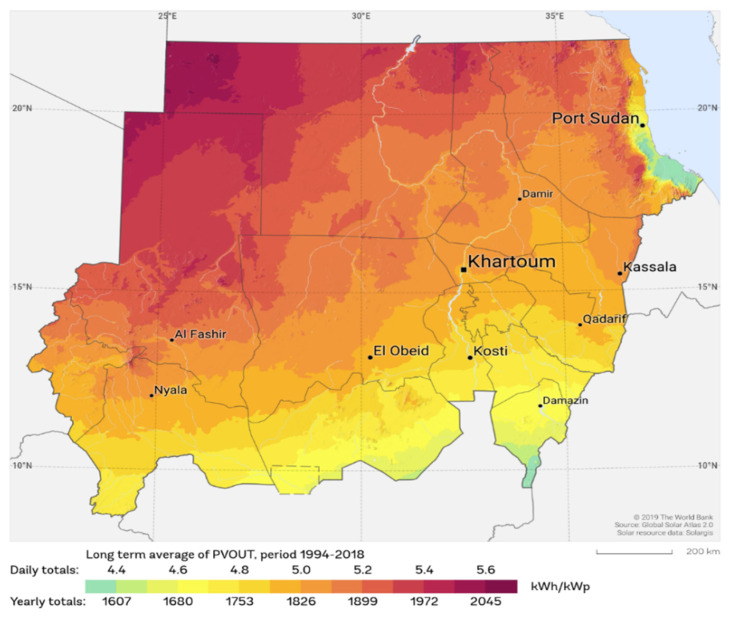
Solar capacity map of Sudan [8].

**Figure 2:**
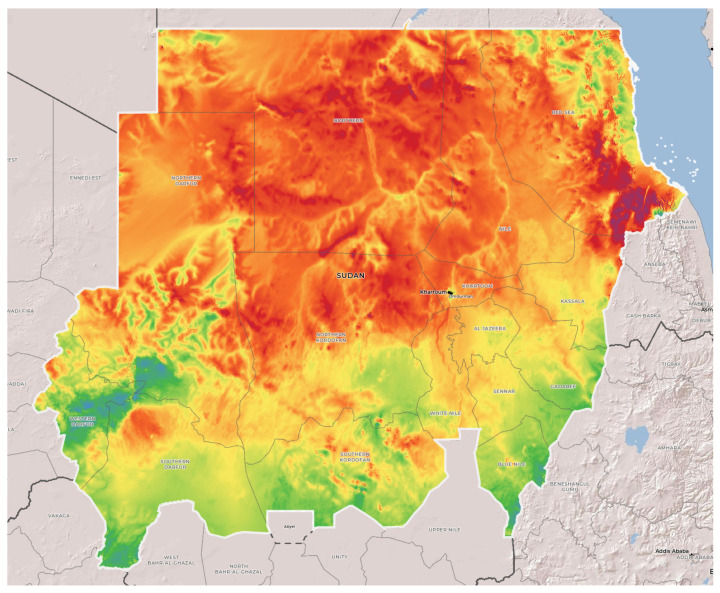
Wind capacity map of Sudan [9].

Sudan has a very high potential for solar and wind energy, as can be seen from Figure 1 [8] and Figure 2 [9]. The wind and solar generation capacity rise from the south to the north. The northern regions tend to have higher solar irradiance and wind speed, whereas higher sunlight is also linked with the arid climate. Therefore, despite having a huge area available for crop cultivation, the arid nature of the terrain limits the potential farming. At the same time, there are huge reserves of underground water which can potentially help to convert the arid land into fertile pastures. The conventional rain-fed irrigation can be converted into controlled irrigation by providing the farmers with opportunities to use underground water when and how they need it. The key challenges in pumping the water out are the limitations of the grid supply in these regions, increasing prices of fuel for diesel generators, the lack of technical capability for the maintenance of diesel generator, and concerns around carbon emissions. The solution to these problems is the use of RES, which can be cheaper, easy to maintain, and emissions free.

As discussed above, the agriculture sector in Sudan can potentially benefit from RES. However, very limited research has been conducted in the use of RES, and in particular the hybridization of RES in the agriculture sector in Sudan. A number of studies simulated the use of renewable energy in Sudan, but only a few considered specific applications to irrigation [10]. Moreover, the studies considering the irrigation application were also aimed at a higher level, and the technical feasibility of the hybrid renewable energy system was not explored at a smaller level [10]. Moreover, all of the previous studies considered the irrigation load as standard, and did not consider the climatic effects and the soil data to evaluate the water requirement of different crops. Therefore, a study is needed to optimize the size of the renewable energy source according to the real crop water requirement.

Considering the above, this paper endeavored to bridge the research gap by presenting a holistic technical feasibility approach to explore the potential of a hybrid renewable energy system for small-farm irrigation applications. The paper systematically develops the approach by considering crop evapotranspiration, crop coefficients, soil types and hydrogeology to determine the load values according to the individual crop water requirement and climatic conditions. The paper is expected to serve as a benchmark study for the design of hybrid renewable energy systems for small-scale irrigation water pumps by considering the individual crop water requirement.

The rest of the paper is organized as follows. Section 2 presents the methodology adopted to develop the approach. Section 3 delineates the case studies, and Section 4 gives the results and discussions of the case studies. Finally, Section 5 gives the conclusion of the paper.

## 2. Methodology

**Figure 3:**
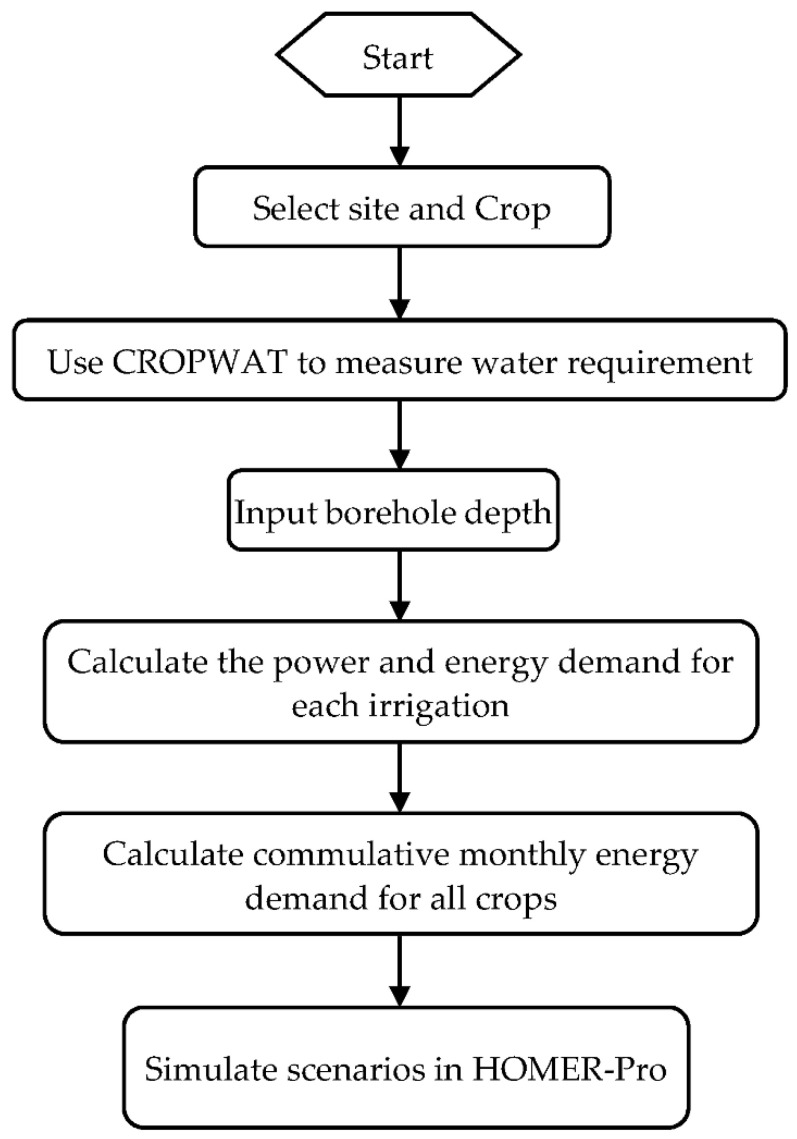
Approach for the design of a hybrid RE system for agriculture.

The optimal design of a hybrid energy system for irrigation requires the development of an integrated framework which can lead to the optimal design solutions. As the aim of the paper is to explore the techno-economic feasibility of the hybrid renewable energy system for irrigation applications, the objective function used for the optimization is the total Net Present Cost (NPC). Using the optimization function, the optimal system configuration is determined whilst considering the design constraints. Figure 3 gives the flow chart for the approach adopted to design the hybrid renewable energy system.

**Figure 4:**
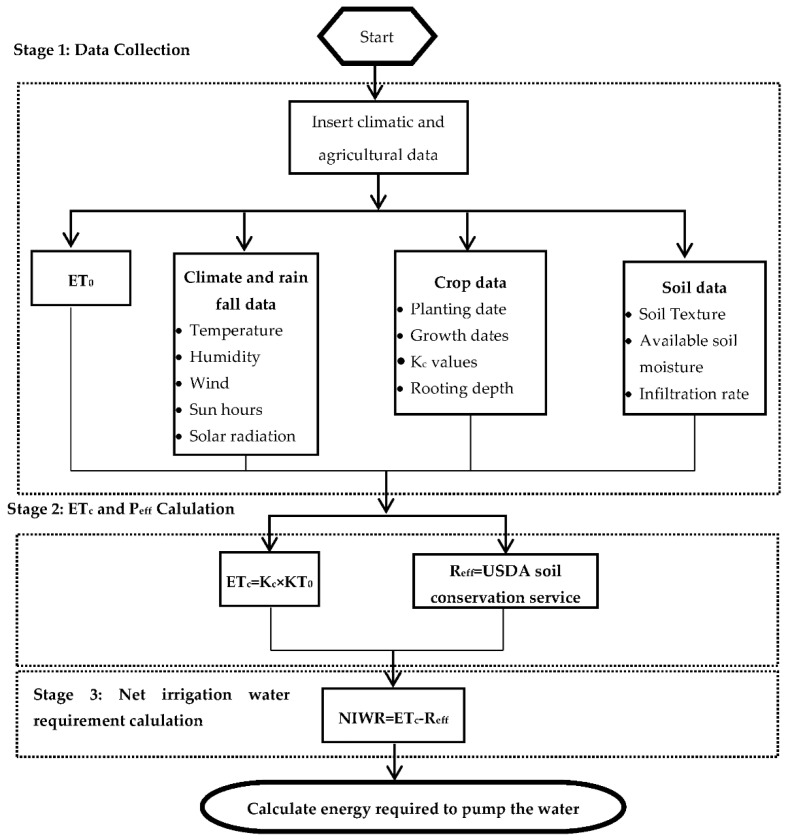
Flow chart for the crop water requirement and energy calculation [12].

When determining the irrigation load, the decisive factor is the crop water requirement. The crop water requirement depends on a multitude of factors, including the crop type, soil type, and climate parameters, etc. Therefore, prior to the consideration of the development of the renewable energy system, the type of crop and site needs to be selected. Once the study sites are selected, the process for the calculation of the crop water requirements is initiated. The crop water requirement evaluation is carried out using CROPWAT software, which is a decision support system developed by the Food and Agriculture Organization (FAO) of the United Nations (UN) [11]. It considers a number of inputs, such as the crop, soil type, climatic data to calculate reference evapotranspiration (ET_0_), crop evapotranspiration (ET_c_), net irrigation water requirement (NIWR), and gross irrigation water requirement (GIWR). In order to calculate the crop water requirement, the methodology presented in Figure 4 was adopted.

Once the site and crop are selected, the climatic and soil data can be collected and used to evaluate the crop water requirement. Apart from the input parameters related to the crop and soil—i.e., crop evapotranspiration, root depth, and type of soil, etc.—climatic data including the temperature and rain is also required to finalize the net irrigation requirement for the crop.

The crop water requirement is converted into an electrical load by considering the borehole depth to design the water-pumping system. The load values of the water-pumping system are converted into energy values by considering the amount of irrigation required for each crop. Finally, monthly energy data are used to design the optimum renewable energy system for the selected site using HOMER software. A detailed study on the crop water requirement is presented in Part I of this paper [12].

The software commonly known as HOMER stands for Hybrid Optimization of Multiple Energy Resources (HOMER), and is used to optimize the use of multiple energy sources. HOMER was developed by the National Renewable Energy Laboratory (RNEL), USA for the technoeconomic modeling, simulation, and optimization of HRESs [13]. HOMER can support the user in designing a hybrid renewable energy system with economic and technical optimization, with optimal sizing to determine the optimal system configuration. HOMER can also carry out a sensitivity analysis to ascertain the sensitivity of a solution to variations in certain parameters.

A number of studies have used HOMER, and it is considered to be the global standard for the optimal planning and design of HRES in all energy sectors [10]. Its application can be found in different sectors, such as radio telecommunication stations [14], desalination plants [15], rural households [16], urban cities [17], and large residential communities [18]. Considering the wider applications of HOMER, it is used in this study to evaluate the techno-economic feasibility of a renewable hybrid energy system for small-scale irrigation purposes in Sudan.

In this paper, 12 sites in Sudan were selected to evaluate the feasibility of the hybrid renewable energy system, and the optimal energy system configuration was simulated for each site. A number of studies using HOMER have considered the application of renewable energy to the irrigation load [10,19,20,21,22]; however, most of these studies consider the irrigation load as a standard periodic load. This practice can potentially result in the over-sizing of the system. On the other hand, the irrigation is calculated either on a daily basis or on a decade basis. This necessarily means that the overall amount of water required in 10 days does not necessarily need to be supplied on the same hour of the day as it is calculated. Thus, the irrigation load is a deferrable load, and has the flexibility to shift the load in time. Considering the above, this paper models the irrigation load as a deferrable load.

**Figure 5:**
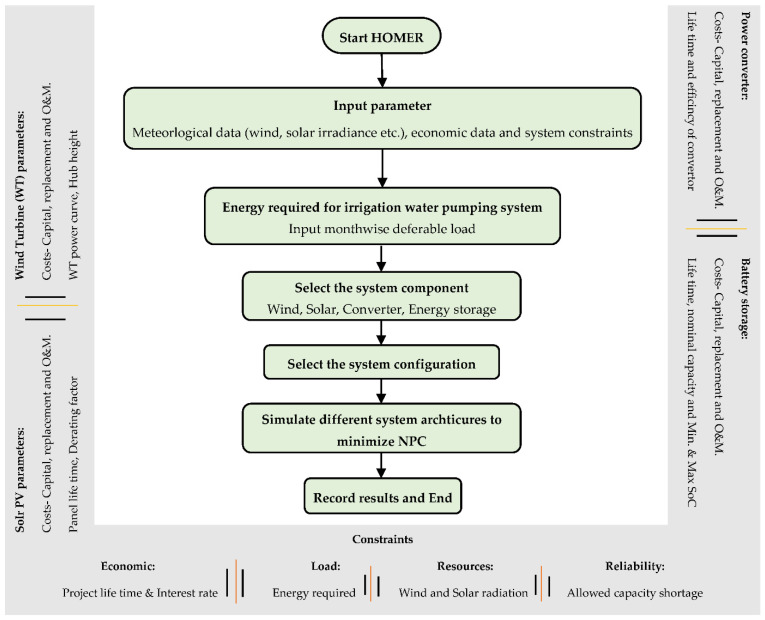
Homer process flow for the optimization of the hybrid renewable system.

Figure 5 shows the flow chart of the process adopted by HOMER to optimize the design of RES. Firstly, all of the input parameters and design constraints—such as meteorological data and system constraints, as well as the design and economic parameters of the system components—are fed to HOMER. Then, the monthly energy required by the water-pumping system at each site is provided as a deferrable load. The energy resources, i.e., wind and solar energy, along with battery storage, are selected, and finally, after selecting the final configuration of the system, the model is simulated with the constraints to find the optimum solution, i.e., the solution with the minimum net present cost.

The process is repeated for each site, and the optimum system architecture is obtained with the economic analysis.

## 3. Case Studies

This paper aims to explore the techno-economic feasibility of a wind–solar hybrid energy system for small-scale irrigation applications in Sudan. Considering the aim, 12 different sites were selected across Sudan. The selected sites were used to evaluate the crop water requirement for three crops, i.e., wheat, cotton and sorghum. According to a special report of the FAO on food in Sudan, most farmers use traditional rain-fed irrigation to irrigate 9 million hectares of land, and this sector serves the largest number of farmers with land between 2 and 50 hectares [23]. As the system was intended for small-scale application, it was assumed that each crop is irrigated in 1 hectare of land, and a total of 3 hectares of land was considered for irrigation purposes at each site. The location of the selected sites is given in Table 1, and the nearest weather station data available in UN FAO CLIMWAT for CROPWAT is also given in the table.

The climate data, soil data and other variables available in the CLIMWAT and CROPWAT were used to calculate the crop water requirement and irrigation requirements. Each site has a different bore hole depth, and the bore hole depth data were used from the British Geological Survey, which holds hydrogeology data [25]. The bore hole depth varied between 40 m and 80 m. An overall analysis of the irrigation requirements at all of the sites showed that the sites located further north require more water due to their arid climate, and thus more energy is required. However, at the same time, the northern sites have more wind and solar energy potential, as is apparent from the wind and solar maps for Sudan.

**Figure 6:**
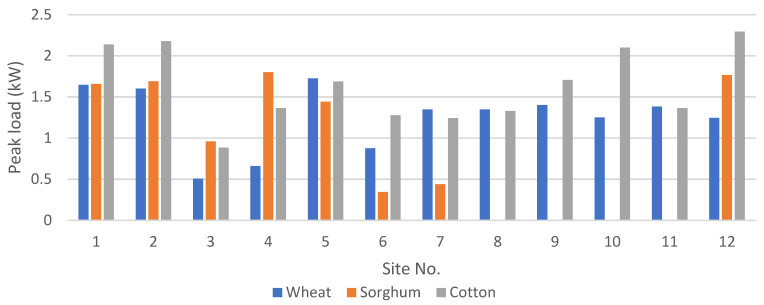
Peak load for each crop at each site.

**Figure 7:**
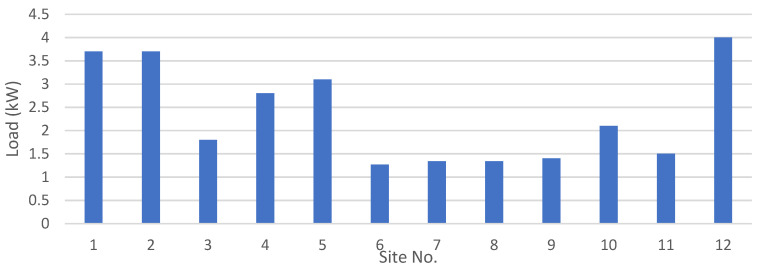
Cumulative peak load for all of the sites [12].

Figure 6 shows the peak load for all 12 sites for each crop individually. It is evident that cotton has a higher peak load, as cotton is a high water-requiring crop. The climate and rain patterns tend to reduce the need for water at different sites for some crops. However, as discussed earlier, the water-pumping system is designed by considering three crops cultivated in a 3 hectare area, and thus the combined load of all three crops is used to design the water-pumping system, which is given in Figure 7. It is not necessary that the peaks of the load coincide, and thus careful consideration should be given to calculate the combined peak in order to avoid the over- or under-sizing of the system. The peak load at each site represents the size of the water pump required at each site in kW.

**Figure 8:**
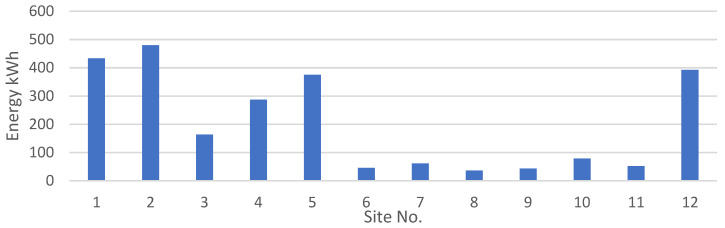
Total energy consumption at each site.

Apart from the peak load, in order to design a hybrid renewable energy system, the energy required is the key parameter. The energy required at different sites in kWh for the entire year is given in Figure 8. It should be noted that the peak loads and energy consumption do not necessarily have a linear relationship. Often, in everyday loads, the peak of the load is correlated with the energy usage, but in the case of agricultural applications it is not compulsory. There is a multitude of factors that affect the energy use of the water-pumping system. These factors primarily govern the crop water requirement, and are discussed above.

**Figure 9:**
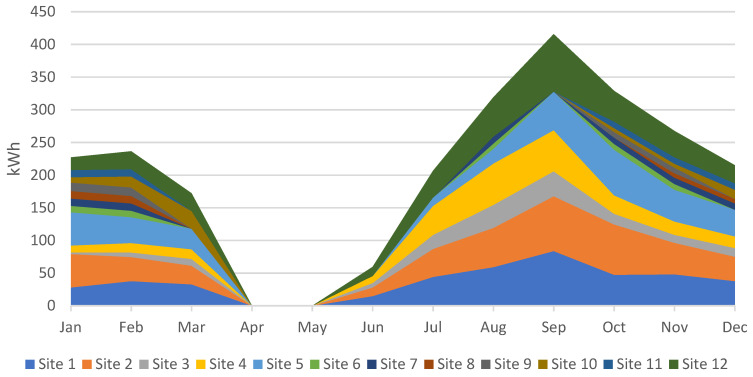
Monthly energy consumption at each site throughout the year.

The energy input required in HOMER should be the monthly energy demand in order to simulate the scenarios for a deferrable load. The monthly energy demand is given in Figure 9, where September is the month with the highest energy demand. Figure 9 presents the energy consumption at each site throughout the year, with the individual contribution of each site in each month. The cumulative effect of the energy consumption is shown by stacking all of the sites. For example, all of the sites show a very high energy demand in September, and thus the overall energy consumption from all of the sites combined is more than 400 kWh in September. A detailed discussion on the cropping patterns and seasons is given in Part I of the paper, which was published separately [12]. A sensitivity analysis of the area for agricultural use and load was carried out, and a direct correlation between the energy use, peak load and the area of agriculture was observed.

The energy required is spread across the entire day, and thus the calculation considers the maximum load based on the assumption that the pump can run any time. Spreading the load delivery over 24 h can potentially benefit the system by reducing the renewable generation size by spreading the load over a longer period of time, and the potential for the hybridization of renewable energy sources is also promoted. It is pertinent to mention that the water pump was designed at peak load, which can only be for a few days during the crop season; the rest of the days, the pump might not need to run all day. However, due to the intermittent nature of the wind and solar PV energy, the water-pumping load was taken as a deferable load.

**Figure 10:**
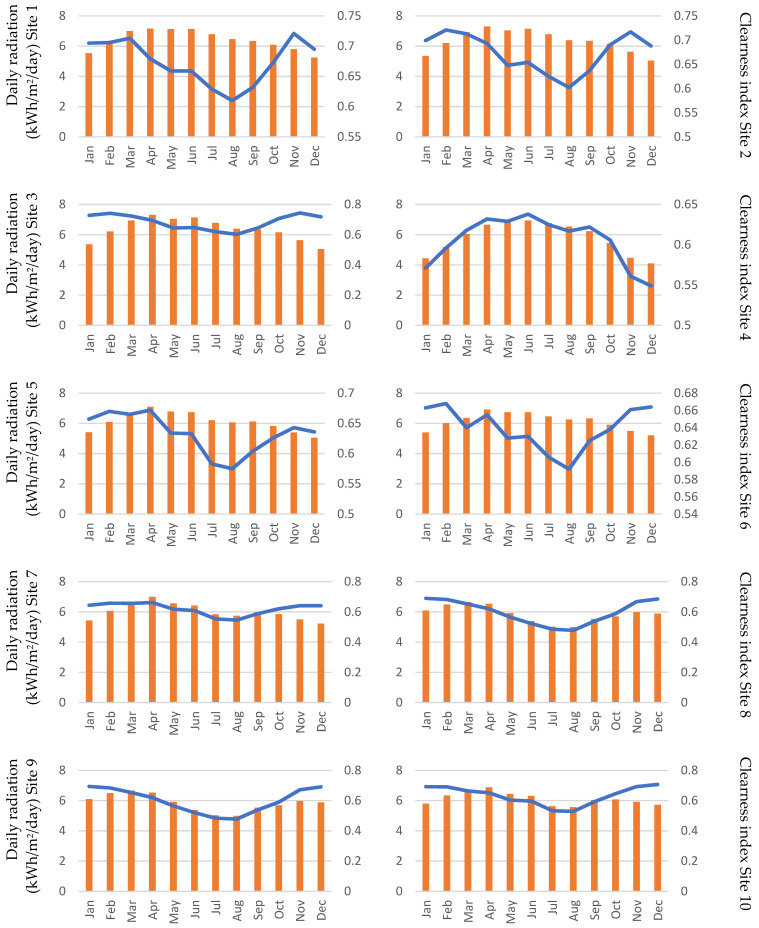

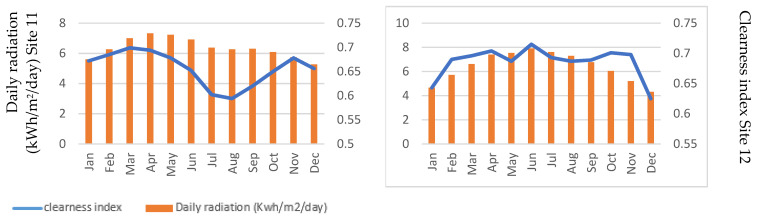
Daily solar radiation and clearness index for the 12 sites (the *x*-axis represents the month of the year, and the primary *y*-axis represents the daily radiation in kWh/m^2^/day; the secondary *y*-axis represents the clearness index).

**Figure 11:**
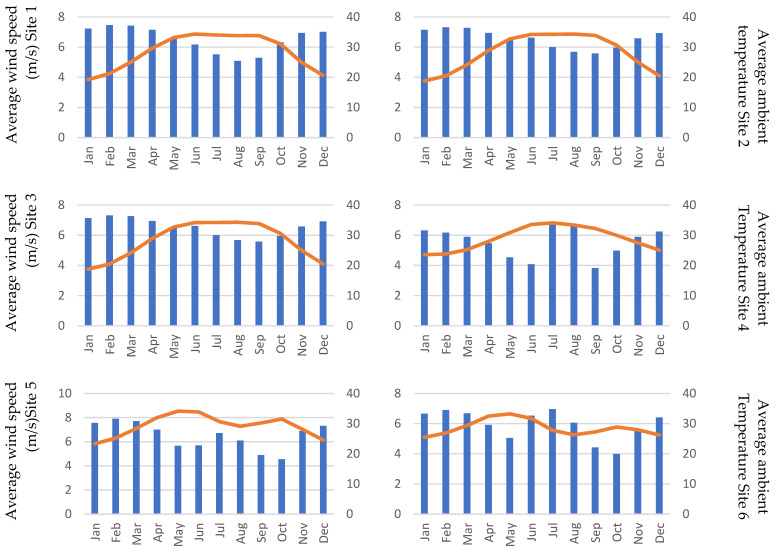

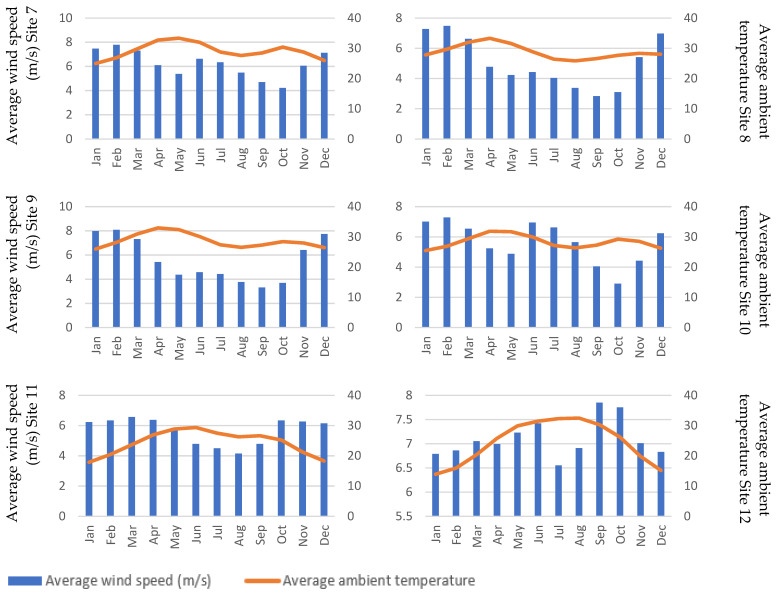
Average wind speed and ambient temperature for the 12 sites (the *x*-axis represents the month of the year, and the primary *y*-axis represents the speed in m/s; the secondary *y*-axis represents the average ambient temperature).

As the overall load and energy calculations were performed, each site was analyzed for wind and solar potential. As is evident from the solar and wind maps of Sudan, a high potential of wind and solar energy is present is Sudan. Figure 10 and Figure 11 show the solar and wind generation potential at each site, as solar irradiance and wind speed. The variation in the wind speed is significant, and during high load periods the wind speed is low, except for sites 11 and 12. Because September is a developing stage of the cotton crop, the energy demand is high, as more water is needed at this stage. The reduction of the wind speed can potentially result in wind being less favorable compared to solar PV, which is not as volatile as wind.

**Figure 12:**
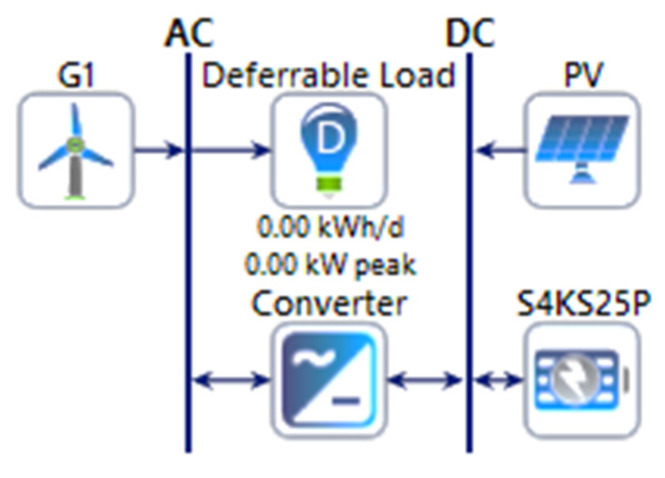
HOMER Pro simulation set up.

As given in Figure 5, a number of constraints are used when designing a hybrid renewable energy system using HOMER Pro. The average wind speed and solar irradiance at each site were the primary constraints. The project life time was set as 25 years, which is a commonly adopted approach considering the fact that such projects are usually government led, and governments tend to have long-term projects. A typical discount rate of 8% was considered. The reliability was set at 100%, i.e., no capacity shortage was allowed, which necessarily means that all of the load demand is met. The price for solar PV was set as $883 per kW, and for a wind generator it was set as $1355, which are global averages [4]. The operation and maintenance (OM) cost for PV was taken as 14 $/kW/year, whereas for wind it was taken as 15 $/kW/year [10]. The Surrette 4KS25P type was used and connected as a central storage system, and the capital cost of one battery was taken as 1250 $, while the OM cost was assumed to be 15 $/year [10]. Once the system constraints and the resources, i.e., wind solar generation resources, battery storage and load, were determined, a simulation setup was developed in HOMER Pro to simulate the case studies for each site, as given in Figure 12.

The simulation setup used in the case studies included wind and solar generation, a battery storage system, and a converter. A schematic diagram of the simulation setup is shown in Figure 12.

## 4. Results and Discussion

The case studies for all 12 sites were simulated using the load data for each site, and the resources constraints determined by climatic data were adopted. As is clearly evident from Figure 10 and Figure 11, almost all of the sites have an abundant amount of wind and solar energy. With the decreasing prices of wind and solar generation technologies, it is expected that due to the intermittent nature of both energy sources and the availability of wind even at night, the hybrid system can potentially perform better both technically and economically. The load calculated considering the crop water requirement was used to determine the optimal system architecture, and the net present cost (NPC) and cost of electricity (COE) were calculated to compare the feasibility of a wind–solar hybrid generation system at each site. Table 2 shows the results of all 12 sites, and the two best architectures are given for each site. Moreover, the excess electricity generated is also given for each site and case.

Only two sites were identified, which resulted in a preference of a hybrid of wind and solar for the purposes of supporting the irrigation load. Despite the optimality of hybrid energy, solar energy was the dominant source of electricity. The hybridization scenario at site 12 showed interesting results compared to all of the other sites, where removing 2 kW wind generation resulted in a 6.5 kW addition in solar generation. This shows the significance of the wind generation, and is an indicator of the fact that despite being almost double in the price, the hybridization of these sources can potentially provide significant benefits.

As the overall cost of a wind turbine tends to be higher than solar PV, the NPC tends to be higher than the solar PV generation at most sites, except site 10 and site 12. The lowest cost of electricity was $0.362, and highest was $1.15. The high costs are the results of the excess electricity generated. The lowest excess electricity was 73.6%, and the highest was 96.2%.

**Figure 13:**
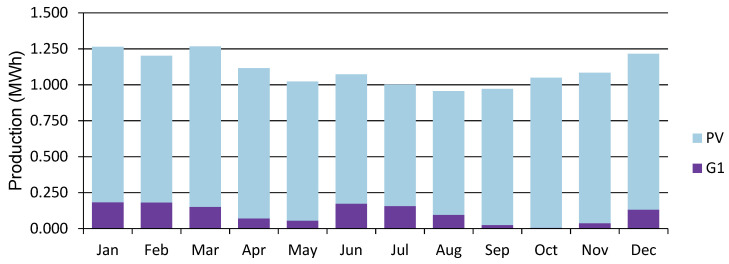
Power generation share at Site 10 (PV represents solar PV generation, and G1 represents wind turbine generation).

**Figure 14:**
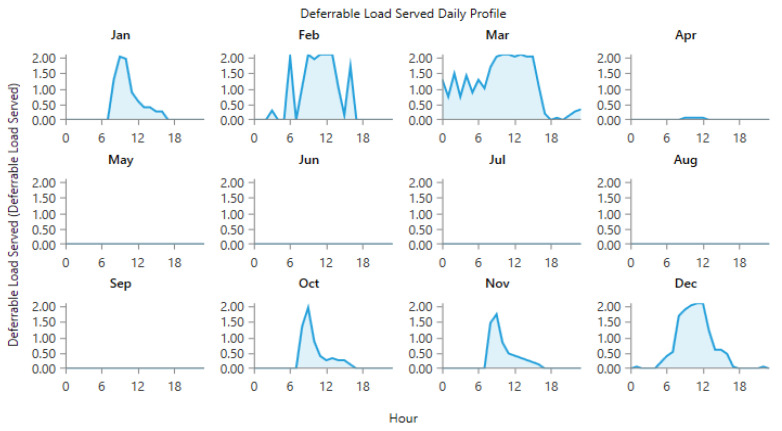
Electric load served (daily profile) at Site 10.

**Figure 15:**
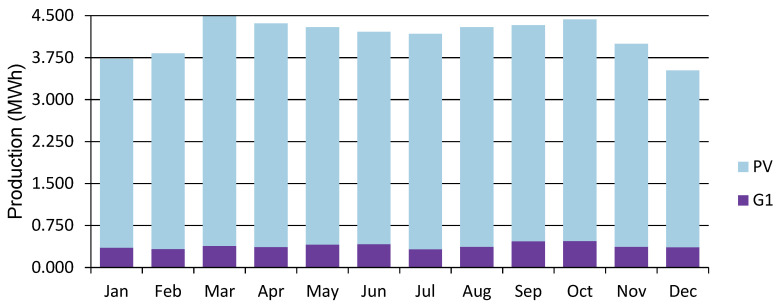
Power generation share at Site 12 (PV represents solar PV generation, and G1 represents wind turbine generation).

**Figure 16:**
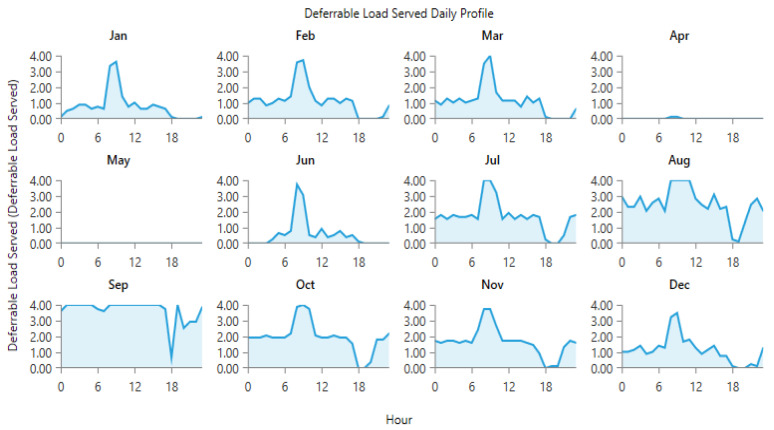
Electric load served (daily profile) at Site 12.

The power generated by the wind and PV generators, and the total electrical load served at site 10 and site 12 is shown in Figure 13, Figure 14, Figure 15 and Figure 16. Figure 13 and Figure 15 show the generation by all of the sources during a single year. It is pertinent to mention that these loads are not operational throughout the year, and the energy demand varies every day and month. However, in order to ensure that all of the load demand is met, the energy system’s size was optimized to provide 100% load serving reliability. Therefore, a large amount of excess energy was observed at all of the sites. As can be seen from the wind profiles, the power production using a wind turbine at site 12 tends to be better than site 10. Moreover, the amount of water and the number of irrigations required at site 12 also contribute to the need of additional power which is provided by the wind generation. As the pump rating is calculated considering the maximum load requirement, Site 12 tends to have a higher demand of electricity and to meet that demand using the rated pump; wind energy provides the extra energy, which can serve the base load to meet the energy needs. The weather patterns, in combination with the soil type, reduce the load significantly for site 10, which is evident from Figure 14. It is evident from Figure 13 and Figure 15 that a great amount of excess energy is produced, which is wasted. Apart from the water-pumping load, the storage system’s annual throughput for site 10 is 440 kWh/year, and for site 12 it is 5383 kWh/year. Moreover, the converter losses for site 10 are 19.6 kWh/year, and for site 12 they are 241 kWh/year. While considering the overall generation, the only energy consumed is through the energy used by the water pump, energy storage throughput and converter losses; a large amount of the energy is surplus, which can potentially be used for other purposes. The energy required at each site for the water-pumping system is given in Figure 8. The excess amount of energy in each case is given in Table 2.

The overall results suggest that the hybridization of wind and solar energy can potentially benefit the system by reducing the need for solar PV multifold. The availability of wind over 24 h can spread the load and reduce the system size; thus, a smaller wind system might be able to replace a bigger solar system; however, the cost of a wind generator compared to solar PV is as yet at a higher level for applications on a small scale. Although the present reduction in cost of on-shore wind generation is promising, wind energy has yet to take the long journey to become feasible for small-scale applications. The application of hybrid energy systems in small scale applications, and particularly in irrigation applications, is not yet feasible in Sudan.

## 5. Conclusions

This paper presents a techno-economic assessment of wind–solar hybrid generation for a water-pumping system for small-scale irrigation projects. A comprehensive framework is presented which systematically develops the energy system using the crop and site data to measure the energy required. The data from 12 sites in Sudan were used to calculate the crop water requirement using FAO CROPWAT software, and the energy need was evaluated using the crop water requirement. HOMER Pro was used for the techno-economic assessment of different architectures, and to evaluate the feasibility of the hybrid energy systems for small irrigation applications. The results suggest that wind energy is suitable at two sites, i.e., site 10 and site 12. Site 12 tends to be more suitable for the hybridization of energy generation compared to all of the other sites. From the case studies, it is apparent that hybrid energy generation is yet not economically feasible due to the higher prices of on-shore wind generation. Therefore, due to the higher costs and technical challenges of wind turbines, and the ease of installation and maintenance of solar PV, solar PV tends to be more suitable for such applications as opposed to a hybrid of wind and solar PV. In conclusion, the wind–solar hybrid energy system for small-scale irrigation applications in Sudan is not yet feasible in most regions.

## Figures and Tables

**Table 1:** Site locations and soil types (Adopted from [24]).

No.	Sample Location	Climate Zone	No. and Name of Nearest Weather Station on CLIMEWAT	Co-Ordinates of Weather Station Long.–Lat.	Soil Type
1	North East of Karima	Arid	54-Karima	31.85–18.55	Poorly graded sand
2	South East of Abuhamad	Arid	63-Abu-Hamed	33.31–19.53	Poorly graded silty sand
3	Osaif	Arid	8-Dongonab	37.13–21.1	Silty sandy Gravel
4	South west of Toker	Arid	59-Tokar	37.73–18.43	Silty Gravelly sand
5	West of Almatama	Arid	38-Khartoum	32.55–15.6	Gravelly Silty sand
6	Kassala	Semi Arid	41-Kassala	36.4–15.46	Silty Clay
7	Elgurashi	Semi Arid	27-Ed-Dueim	32.33–14	Silty Clay
8	Kadugli	Semi Arid	9-Kadugli	29.71–11	Sandy Silty Clay
9	Almujlad	Semi Arid	7-Babanusa	27.81–11.33	Silty Clay
10	Algadarif	Semi Arid	34-Gadaref	35.4–14.03	Silty Clay
11	Kutum North of Darfur	Semi Arid	33-Kutum	24.66–14.2	Silty Clay
12	Wadi-Halfa	Arid	3-Wadi-Halfa	31.48–21.01	Poorly graded sand

**Table 2:** Results of HOMER Pro for 12 Sites.

Site No.	Best Case	Architecture	PV (kW)	WT (kW)	Batt (Nos.)	Conv (kW)	NPC ($)	COE ($)	Excess Electricity (%)
1	i	PV-Battery/Converter	31.4	0	21	16.4	70,987	0.417	75.1
1	ii	PV-WT-Battery/Converter	31.2	1	21	15.9	73,010	0.429	75.8
2	i	PV-Battery/Converter	32.9	0	20	9.35	68,183	0.362	73.6
2	ii	PV-WT-Battery/Converter	32.1	1	19	9.43	68,302	0.362	73.9
3	i	PV-Battery/Converter	14.3	0	10	4.88	31,960	0.496	73.6
3	ii	PV-WT-Battery/Converter	13.8	1	9	4.99	32,450	0.504	73.9
4	i	PV-Battery/Converter	32.3	0	14	7.62	57,485	0.508	82.3
4	ii	PV-WT-Battery/Converter	28.5	1	15	7.71	57,729	0.510	80.6
5	i	PV-Battery/Converter	24.7	0	17	10.1	55,516	0.377	71
5	ii	PV-WT-Battery/Converter	25.2	1	16	14.7	58,803	0.399	72.8
6	i	PV-Battery/Converter	9.11	0	1	0.130	10,832	0.609	91.8
6	ii	PV-WT-Battery/Converter	8.85	1	1	0.260	11,411	0.641	92.3
7	i	PV-Battery/Converter	14.8	0	1	0.130	16,595	0.687	93
7	ii	PV-WT-Battery/Converter	15.1	1	1	0.260	19,396	0.803	93
8	i	PV-Battery/Converter	4.03	0	2	15	13,234	0.939	83
8	ii	PV-WT-Battery/Converter	2.79	1	2	18.3	15,750	1.12	79.5
9	i	PV-Battery/Converter	11.9	0	1	0.680	13,612	0.814	93.8
9	ii	PV-WT-Battery/Converter	11.5	1	1	0.521	15,811	0.945	93.9
10	i	PV-WT-Battery/Converter	6.48	1	6	4.46	20,125	0.658	81
10	ii	PV-Battery/Converter	7.53	0	7	4.68	20,386	0.666	81.9
11	i	PV-Battery/Converter	21.4	0	1	0.13	23,222	1.15	96.2
11	ii	PV-WT-Battery/Converter	21.4	1	1	0.26	25,723	1.28	96.3
12	i	PV-WT-Battery/Converter	22.8	2	17	9.71	58,312	0.379	72.5
12	ii	PV-Battery/Converter	29.3	0	17	11.1	61,656	0.400	76.1
